# The procurement and supply chain strengthening project: improving public health supply chains for better access to HIV medicines, Uganda 2011–2016

**DOI:** 10.1186/s40545-022-00467-3

**Published:** 2022-10-27

**Authors:** Sowedi Muyingo, David Etoori, Paul Lotay, Samuel Malamba, James Olweny, King Keesler, Steven Wiersma, Pito Jjemba, Rashid Settaala

**Affiliations:** 1 Medical Access Uganda Limited, Kampala, Uganda; 2 London School of Hygiene and Tropical Medicine, London, UK; 3 Uganda Virus Research Institute-National Reference Laboratory, Plot 51-59 Nakiwogo Road, P.O. Box 49, Entebbe, Uganda; 4 U.S. Centers for Disease Control and Prevention, Kampala, Uganda

**Keywords:** HIV, Supply chain, Stock-outs, Stock management

## Abstract

**Background:**

With countries moving towards reaching the UNAIDS 90-90-90 goal to achieve HIV epidemic control, there are going to be an unprecedented number of persons who will need to be tested, treated, and regularly monitored for viral suppression. However, most of the countries with the greatest burden of HIV/AIDS experience regular stock outages which could be detrimental to reaching these targets. ART and other commodities such as HIV test kits and laboratory supplies need to be readily and consistently available to achieve these targets. The main objective was to improve access to HIV/AIDS related commodities and strengthening institutional capacity for the management of HIV/AIDS logistics services through the MAUL procurement and supply chain strengthening project (PSSP) that rolled out four interventions on mentorship and support supervision, stock level monitoring, spatial visualization of stock indicators using GIS, and using WhatsApp to submit order reports as photo images.

**Methods:**

Medical Access Uganda Limited, a private-not-for-profit supply chain management company in Uganda, implemented these interventions as part of a procurement and supply chain strengthening project (PSSP). These interventions were evaluated using performance monitoring indicators from 2011–2016. We tested for the significance in the change in scores of performance monitoring indicators using the test for difference in proportions. Health facilities were scored on 6 categories and accredited as bronze, silver or gold based on their total scores. Kaplan–Meier estimates were computed for time to silver, and gold ranking and univariate and multivariate Cox proportional hazards models were computed for time to gold ranking.

**Results:**

We observed a significant reduction in reported stock-outs from 46 to 4% (*p* < 0.001) in the analysis period. Accurate stock card inventory rose from 79 to 91% (*p* < 0.001); adequate stock levels rose from 54 to 71% (*p* = 0.002) and stock reporting rates from 91 to 100% (*p* < 0.001). The stock order fill rate improved from a high of 93% to 97% (*p* = 0.375). Patient load (medium vs low adjusted hazard ratio (aHR): 2.19, *p* = 0.026; high vs low aHR: 2.97, *p* = 0.034) and number of support supervision visits (6–10 aHR: 3.33, *p* = 0.024; > 10 aHR: 5.78, *p* = 0.003) were associated with better stock management ranking scores.

**Conclusions:**

Improvements in supply chain management in countries committed to achieving the 90-90-90 goals are crucial to achieving HIV epidemic control. Health system strengthening and mentorship investments in Uganda were feasible and are essential for sustainable disease control efforts.

## Background

With countries moving towards achieving the 90-90-90 goal to achieve HIV epidemic control, the number of patients on antiretroviral therapy (ART) is increasing steadily [1, 2]. However, a question still remains on the ability of the prevailing systems to reach, treat and suppress the prevalent cohort of 25.6 million people living with HIV (PLHIV) in sub-Saharan Africa (SSA) [3]. To achieve the ambitious goal of population viral load suppression, it is critical to ensure timely supply of ART. In SSA, many countries have weak supply chain management (SCM) systems [4].

Despite unprecedented investment in SCM for HIV since 2010, many SSA countries still report widespread stock-outs of essential medications. A pilot study in Ethiopia in 2015, for example, reported 21% of health centers and hospitals having no ART medicines and 33% of health posts having no rapid HIV tests [5]. Indeed, a dysfunctional supply chain impedes the whole HIV test and treat cascade in terms of number of people tested, numbers of people testing positive who can start ART, and risk of disease progression for patients on ART due to supply ruptures. This adversely impacts population HIV suppression and, moreover, may have far reaching implications in terms of HIV drug resistance as a result of suboptimal drug adherence due to ART stock-outs [6].

Many of the problems in SCM can be categorized as either micro-level (organizational) or macro-level (nationwide constraints, i.e., road networks, accessibility demographics, national medicine policies). At the micro-level the supply chain is made up of several steps such as procurement, warehousing and distribution, there are a potential number of areas to address in order to improve SCM. Whereas a lot has been done to improve the “first mile” (quantification, ordering, production, shipping, customs clearance, and miscellaneous in-country requirements) in national SCM; this on its own is not sufficient. While we find that nationwide stock-outs are rare, it is common at the facility and community level [7, 8]. We believe that more emphasis needs to be placed on the “last mile” (distribution from warehouses to the final destination in the health facilities and community) of the micro-level supply chain to achieve the UNAIDS HIV targets.

SCM is increasingly difficult in SSA due to diversity of ART medicines, the large numbers of health facilities, and the reach into remote rural areas [9, 10]. Archaic stock management practices and poor national infrastructure further exacerbate the situation. For example, wastage due to expiry and loss due to damage and mishandling are quite common and unacceptable given the need and the costs of these medications. Furthermore, a lack of health workers in SSA, including fewer than one pharmacist per 10,000 population is a barrier to achieving epidemic control [11, 12]. Positive patient-level interventions may not achieve favorable treatment outcomes if they are not combined with micro-level SCM interventions [13].

In 1998, Medical Access Uganda Limited (MAUL) was started as a pilot project of the Joint United Nations Program on HIV/AIDS in sub-Saharan Africa. Since then, MAUL has proven groundbreaking and has developed and implemented public health SCM solutions to ensure access to quality HIV healthcare for the people of Uganda. MAUL is a not-for-profit Ugandan organization that specializes in logistics and supply chain technical assistance and service provision in SSA.

We identified two broad categories of constraints in SCM in Uganda that needed to be addressed: endogenous (within MAUL) and exogenous (stemming from health facility (HF) level) operational constraints. Inadequate use of stock data and expiry dates, as well as a lack of adequate communication between facilities and with the regional and central distribution stores meant that we were missing out on stock redistribution opportunities to prevent stock-outs. Lack of a computer, reliable power source, or reliable internet connectivity were communication barriers in a number of health facilities which led to delays in placing ART and HIV test kit orders. As a result, order reporting rates were below 80% and order timeliness (defined as an order received before the 5th of every month) was 68% in 2010.

Here we present the experience of MAUL procurement and supply chain strengthening project (PSSP) operations in Uganda, the interventions used, and how these interventions improved SCM. The aim of this study was to improve access to HIV/AIDS related commodities and strengthen institutional capacity for management of HIV/AIDS logistics services through the MAUL PSSP that rolled out four major interventions which included mentorship and technical health systems strengthening (MaTHSS) support supervision; field reporting on stock level tracking (FROST); spatial visualization of stock indicators using GIS; and using WhatsApp to submit order reports as photo images.

The specific objectives of the study were: (a) to assess the effect of MaTHSS support supervision on the overall SCM performance of health facilities and to determine independent predictors of performance improvement; (b) to estimate the time taken by a health facility and the cumulative probability of achieving a higher performance status; (c) to assess the effect of FROST and GIS interventions on stock redistributions, the proportion of health facilities with adequate stock levels of indicator commodities and percentage stock levels; and (d) to estimate the effect of submitting WhatsApp messenger order reports as photo images on facility reporting as well as customer order fill rates.

## Methods

### Setting of the study

MAUL was awarded a 5-year PEPFAR grant in 2011, through the U.S. Department of Health & Human Services (HHS) under the U.S. Centers for Disease Control & Prevention (CDC) to implement the Procurement & Supply Chain Strengthening Project (PSSP). The main goal was to support procurement and logistics management services for HIV/AIDS‐related commodities—including ART, medicines for Opportunistic Infections (OIs), laboratory reagents, equipment and consumables to Private-Not‐For‐Profit (PNFP) Health Facilities and strengthen institutional capacity for management of HIV/AIDS logistics services. Project funding increased from USD 7.6 million in 2011–2012, to USD 46 million in 2012–2013 and USD 60 million in 2015–2016. PSSP provided ARVs to over 250,000 patients in 216 participating health facilities across 62 districts in Uganda in 2015–2016.

Overtime, we implemented four major interventions to address these constraints at the health facility level, including Mentorship and Technical Health Systems Strengthening (MaTHSS) supportive supervision model in 2011–2012, the Field Report on Stock Tracker (FROST), Geographic Information Systems (GIS), and WhatsApp^®^ Messenger in 2014 in an attempt to improve SCM indicators.

### Interventions to address the gaps

#### The Mentorship and Technical Health Systems Strengthening (MaTHSS) supportive supervision model

Starting in 2012 and implemented in 198 health facilities, we combined on-site mentorship, training on Logistic Management Information Systems (LMIS) and SCM and support supervision to achieve defined goals. Regional Field Support Officers (RFSOs) performed on-site mentorship of health facility staff during support supervision visits. Trainings were performed by a team comprising supply chain technical officers, monitoring and evaluation (M&E), LMIS and field operations teams.

Health facilities were assessed using the Support Supervision Monitoring Tool (SSMT) that scored them in six broad categories: (1) stock management, (2) product organization, (3) dispensary, laboratory and store management, (4) dispensing aids and tools, (5) ordering and reporting, and (6) expiry tracking. Each category had a maximum score possible, and the categories were added up to give a maximum total score. Facilities were graded in each category and their scores added up to give a facility total score. Facilities were then categorized—based on their total score as a percentage of the maximum total score possible—as unranked (< 50%), bronze ranking (50–69%), silver ranking (70–90%) and gold ranking (> 90%). Direct feedback was provided to health facilities staff, and senior management to ensure sustainable improvement in overall performance. Facilities could graduate to a new category if they implemented recommended actions and scored in a higher category on two–three consecutive visits.

Facilities were also assessed using three internal performance monitoring indicators that recorded the number of health facilities personnel trained in LMIS and SCM, and the number of support supervision visits monthly, quarterly, and annually.

#### Field Report on Stock Tracker (FROST)

In 2014, we developed a field-based tool (FROST) to monitor stock levels at 191 health facilities receiving ART medicines and laboratory supplies. This field-based tool enabled RFSOs and logisticians to manage commodities by visualizing ART stock levels at all health facilities.

Health facility consumption rates and physical counts for HIV commodities were updated into FROST monthly. The tool generated available months of stock, thereby facilitating commodity decision-making for borrowing or lending of HIV commodities to and from nearby health facilities.

#### Geographic Information Systems (GIS)

In 2014, we utilized a four-stage approach of linking Logistics Management Information Systems (LMIS) to GIS. Logistics staff members were trained in GIS spatial and temporal analyses. GIS coordinates were then collected from 216 health facilities. Confirmation of coordinates was done using mobile-phone reconnaissance and merged into a central level LMIS database, to form crosswalk tables. GIS data were used together with FROST data to spatially visualize stock-on-hand, stock-outs and other important stock indicators.

#### WhatsApp^®^

For each health facility with communication problems, we identified a health worker with a smart phone. WhatsApp^®^ Messenger was installed on the smart phone and health workers trained on how to take pictures of order reports to be forwarded to the warehouse. The image was transcribed into an electronic version and processed for resupply. Acknowledgment of receipt for all orders received at the warehouse was done immediately by warehouse staff.

### Performance assessment

All the interventions were assessed following a pre-specified Performance Monitoring Plan (PMP) a set of performance indicators developed in-house to evaluate our achievement of specified goals. These indicators are broken down into seven major categories: (1) product selection, (2) forecasting and quantification, (3) procurement, (4) storage and warehousing, (5) order processing, (6) inventory management and facility reporting, and (7) supervision and training.

### Variables and definitions

For purposes of assessing performance, the following six indicators were used:

A. Percentage of facilities with adequate stock levels of indicator commodities to ensure near-term continuous product availability (*total number of health facilities reporting stock levels of indicator commodities within minimum/maximum range divided by total number of health facilities*).
B. Inventory accuracy for on-hand inventory at the end of the reporting period (*number of health facilities whose physical count tallies the stock card record divided by total number of health facilities visited*).
C. Average percentage stock in levels at the facilities (*total number of health facilities that did not report a stock-out for any indicator commodity divided by the total number of health facilities that reported in a cycle*).
D. Order fill rate in terms of customer receipts (*total product ordered minus total product received by health facilities divided by total number of products ordered by health facilities*). We also assessed timeliness, completeness, and efficiency of the distribution system.
E. Facility reporting rates (*number of health facilities that submitted complete LMIS reports according to the defined reporting schedule divided by the total number of health facilities reporting*).
F. Number of health facility personnel trained in LMIS or SCM and number of support supervision visits conducted in health facilities.

### Data management and statistical analysis

For this analysis, data were aggregated from several data sources including excel spreadsheets and electronic databases developed in-house. SSMT data entry was performed using Epidata 3.1 [14]. Data cleaning and consistency checks were performed on aggregated data to ensure correctness.

For health facility characteristics, categorical variables were described using frequency statistics and proportions and for continuous variables we reported median and interquartile range. For changes in performance monitoring indicators, we tested for the significance in the change in scores using the test for difference in proportions. Kaplan–Meier estimates were computed for time to silver, and gold ranking and univariate and multivariate Cox proportional hazards models were computed for time to gold ranking. All analyses were done using Stata 14 [15].

## Results

**Fig. 1:**
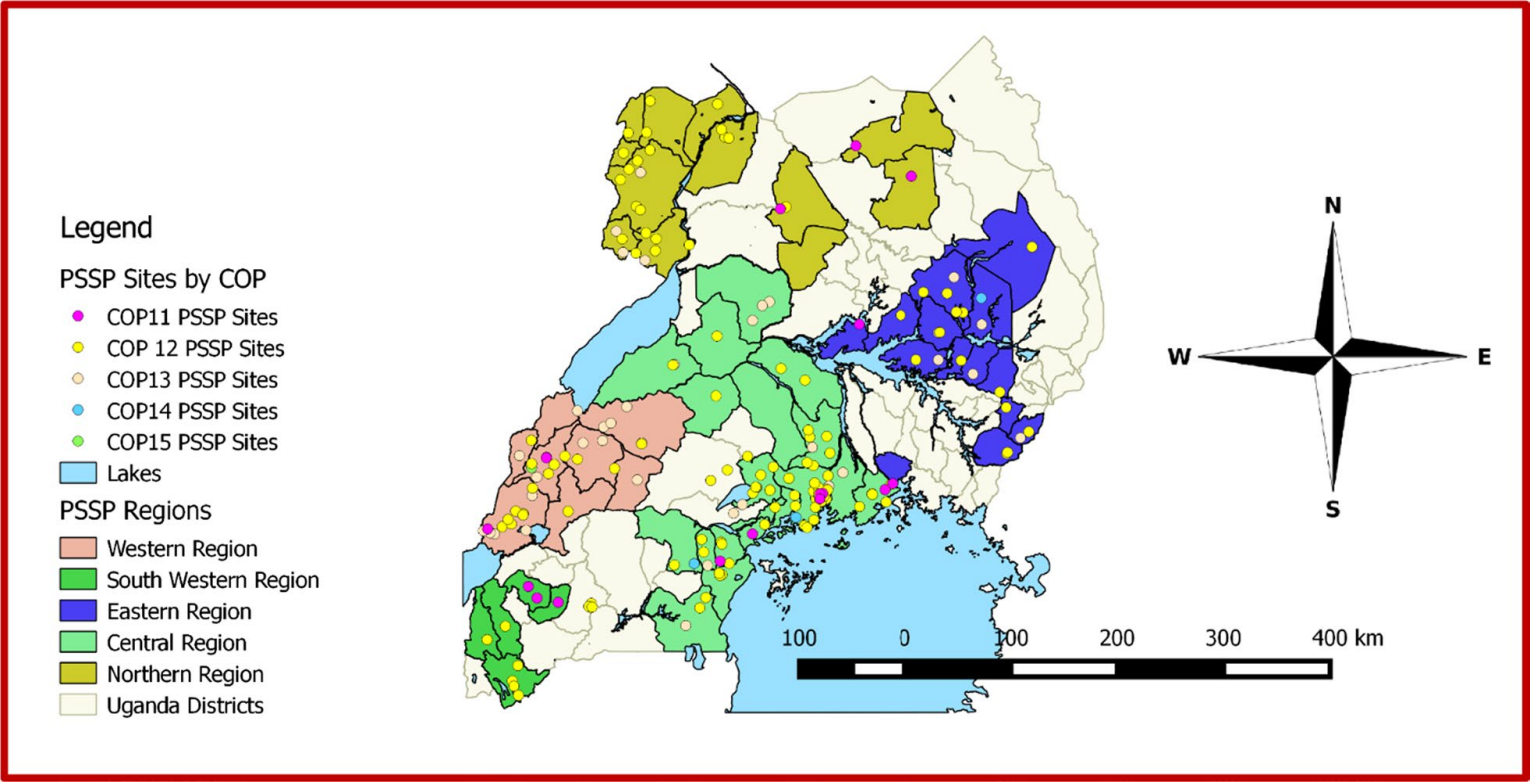
Map of Uganda showing the location of the health facilities and their start year

Starting with 152 HF in 2013, by the end of the analysis period in 2016, we supplied a total of 216 HF which facilities were a median distance of 220 km (IQR: 68, 363) from MAUL headquarters in Kampala, the capital city of Uganda (Fig. 1). These HF were run by 12 PEPFAR-funded implementing partners. One HF (0.5%) was classified as a level 2 health center (HC II), 136 (63%) as HC III, 11 (5.1%) as HC IV, 37 (17.1%) as hospitals and 31 (14.3%) as special clinics. A total of 102 (47.2%) were located in central Uganda, 29 (13.4%) in the East, 31 (14.4%) in the North, 24 (11.1%) in the Southwest and 30 (13.9%) in the West. Median patient load in the HF was 245.5 (IQR: 79, 1073). One HF has since been closed (Table 1). The average baseline SSMT score, and patient load varied by region (Table 2).

**Table 1:** Characteristics of health facilities supported to improve supply chain management in Uganda by improvement rank

	Assessed	Unranked	Bronze	Silver	Gold
Total	198	18	6	109	83
Region (*n* = 198)	*n* (%)	*n* (%)	*n* (%)	*n* (%)	*n* (%)
Central	94 (47.5)	8 (44.4)	5 (83.3)	54 (49.5)	35 (42.2)
Eastern	26 (13.1)	3 (16.7)	1 (16.7)	19 (17.4)	6 (7.2)
Northern	29 (14.6)	2 (11.1)	0 (0)	22 (20.2)	7 (8.4)
South Western	23 (11.6)	1 (5.6)	0 (0)	3 (2.8)	20 (24.1)
Western	26 (13.1)	4 (22.2)	0 (0)	11 (10.1)	15 (18.1)
Distribution sector (*n* = 198)					
Sector 1	42 (21.2)	2 (11.1)	1 (16.7)	35 (32.1)	6 (7.2)
Sector 2	16 (8.1)	1 (5.6)	0 (0)	9 (8.3)	7 (8.4)
Sector 3	32 (16.2)	3 (16.7)	1 (16.7)	21 (19.3)	10 (12.0)
Sector 4	73 (36.9)	10 (55.6)	2 (33.3)	24 (22)	47 (56.6)
Sector 5	35 (17.7)	2 (11.1)	2 (33.3)	20 (18.3)	13 (15.7)
HF level (*n* = 198)					
HC II	1 (0.5)	0 (0)	1 (16.7)	0 (0)	0 (0)
HC III	120 (60.6)	16 (88.9)	3 (50)	74 (67.9)	43 (51.8)
HC IV	11 (5.5)	0 (0)	0 (0)	4 (3.7)	7 (8.4)
Hospital	35 (17.7)	2 (11.1)	0 (0)	16 (14.7)	19 (22.9)
Special clinic	31 (15.7)	0 (0)	2 (33.3)	15 (13.8)	14 (16.9)
Patient load (*n* = 194)					
Low volume (< 100)	55 (27.8)	0 (0)	0 (0)	42 (38.5)	13 (15.7)
Medium volume (100–999)	87 (43.9)	0 (0)	1 (16.7)	43 (39.4)	43 (51.8)
High volume (≥ 1000)	52 (26.3)	0 (0)	1 (16.7)	24 (22)	27 (32.5)
Missing	4 (2.0)	18 (100)	4 (66.7)	0 (0)	0 (0)
Support supervision visits (*n* = 198)					
≤ 5	38 (19.2)	0 (0)	6 (100)	25 (22.9)	7 (8.4)
6–10	113 (57.1)	0 (0)	0 (0)	69 (63.3)	44 (53)
> 10	47 (23.7)	0 (0)	0 (0)	15 (13.8)	32 (38.6)
Missing (no visits)	0 (0)	18 (100)	0 (0)	0 (0)	0 (0)
Status (*n* = 198)					
Active	194 (98.0)	11 (61.1)	6 (100)	108 (99.1)	80 (96.4)
Closed	0 (0)	1 (5.6)	0 (0)	0 (0)	0 (0)
Not active	4 (2.0)	6 (33.3)	0 (0)	1 (0.9)	3 (3.6)
HF category (*n* = 216)					
Ranked	194 (98.0)	0 (0)	2 (33.3)	109 (100)	83 (100)
Ranked scores missing	4 (2.0)	0 (0)	0 (0)	0 (0)	0 (0)
Not ranked (closed)	0 (0)	1 (5.6)	4 (66.7)	0 (0)	0 (0)
Not ranked (not assessed)	0 (0)	17 (94.4)			
Start year (*n* = 198)					
2013	152 (76.8)	0 (0)	0 (0)	84 (77.1)	68 (81.9)
2014	36 (18.2)	0 (0)	2 (33.3)	21 (19.3)	13 (15.7)
2015	7 (3.5)	0 (0)	1 (16.7)	4 (3.7)	2 (2.4)
2016	3 (1.5)	0 (0)	3 (50)	0 (0)	0 (0)
Missing	0 (0)	18 (100)	0 (0)	0 (0)	0 (0)
Preliminary assessment score (*n* = 194)^§^					
< 50	13 (6.6)	0 (0)	2 (33.3)	8 (7.3)	3 (3.6)
50–69	44 (22.2)	0 (0)	0 (0)	26 (23.8)	18 (21.7)
70–89	106 (53.5)	0 (0)	3 (50)	62 (56.9)	41 (49.4)
≥ 90	31 (15.7)	0 (0)	1 (16.7)	10 (9.2)	20 (24.1)
Missing	4 (2.0)	18 (100)	0 (0)	3 (2.8)	1 (1.2)

^§^18 health facilities were not assessed as of June 2016

Continuous variables

Distance from HQ to health facility served (km) (number of sites = 204); median (IQR) 220 (68, 363)

Patient numbers in a health facility served (number of sites = 194); median (IQR) 245.5 (79, 1083)

Characteristics of health facilities analyzed on the interventions put in place to improve supply chain management in Uganda

**Table 2:** Characteristics of health facilities supported to improve supply chain management in Uganda by region

Region	Central	Eastern	Northern	Southwestern	Western
	Mean (S.D)	Mean (S.D)	Mean (S.D)	Mean (S.D)	Mean (S.D)
Average preliminary SSMT score (%)	74.3 (13.8)	67.8(16.3)	80.5(10.5)	87.8 (6.4)	70.6 (17.8)
Average distance from regional office (km)	74.4 (64.5)	88.4 (63.8)	118.0 (94.8)	107.7 (74.2)	58.8 (40.5)
Average distance from headquarters (km)	74.4 (64.5)	271.7 (91.4)	455.2 (62.9)	361.1 (59.0)	301.6 (61.9)

	*N* (%)	*N* (%)	*N* (%)	*N* (%)	*N* (%)
Patient load (*n* = 194)					
Low (< 100)	21 (20.6)	10 (34.5)	15 (48.4)	5 (20.8)	4 (13.3)
Medium (100–999)	43 (42.2)	6 (20.7)	8 (25.8)	10 (41.7)	20 (66.7)
High (≥ 1000)	27 (26.5)	9 (31.0)	6 (19.4)	8 (33.3)	2 (6.7)
Missing	11 (10.8)	4 (13.8)	2 (6.5)	1 (4.2)	4 (13.3)
Health facility level (*n* = 216)					
HC II	1 (0.98)	0(0)	0(0)	0(0)	0(0)
HC III	60 (58.8)	17 (58.6)	23 (74.2)	11 (45.8)	25 (83.3)
HC IV	5 (4.9)	1 (3.5)	0 (0)	3 (12.5)	2 (6.7)
Hospital	14 (13.7)	6 (20.7)	7 (22.6)	7 (29.2)	3 (10)
Special clinic	22 (21.6)	5 (17.2)	1 (3.2)	3 (12.5)	0 (0)

Characteristics of the 216 health facilities supported to implement the interventions put in place to improve supply chain management in Uganda by region

### MaTHSS

Of the 216 HF supported, 198 (91.7%) HF received a preliminary SSMT score as of June 2016. Facility scores were as follows: < 50% got no rank; 50–69% was ranked bronze, 70–89% ranked as silver and ≥ 90% ranked as gold. At baseline, a total of 13 HF (6.6%) scored < 50%, 44 (22.2%) scored 50–69%, 106 (53.5%) scored 70–90%, 31 (15.7%) scored > 90% and 4 (2%) had missing scores. Currently 83 (41.9%) are categorized as gold, 109 (55.1%) as silver and 6 (3.0%) as bronze (Table 1).

Once a HF received a preliminary SSMT score, it took a median of 7.6 months (IQR: 4.1, 13.2) to achieve silver status and a further 19.2 months (IQR: 12.2, 24.3) to achieve a gold status.

**Fig. 2:**
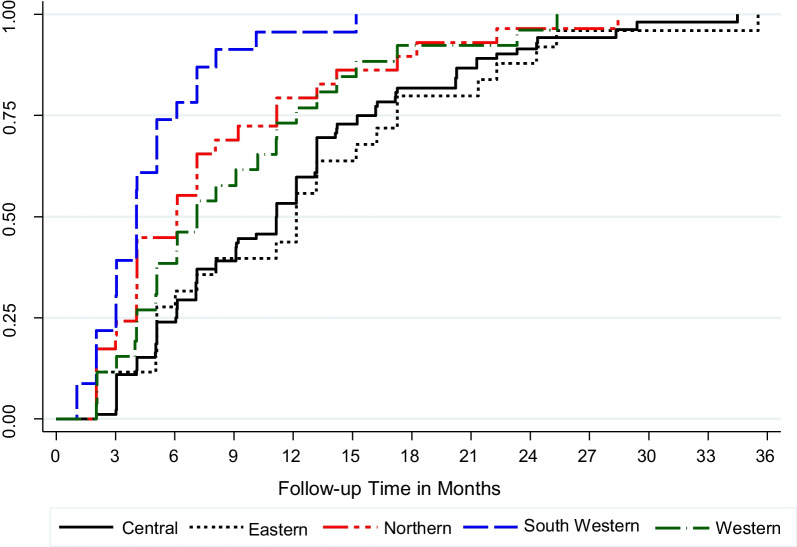
Kaplan–Meier curve comparing time to silver ranking of health facilities stratified by region, Uganda 2011–2016. Kaplan–Meier curve comparing time to silver ranking of health facilities evaluated on health system strengthening in the Procurement and Supply Chain Strengthening Project (PSSP) stratified by region, Uganda 2011–2016

Kaplan–Meier estimates showed cumulative probability of achieving silver status as 100% at 35.5 months. We observed a significant difference in time to silver status by region: Central and Eastern 35.5 months, Northern region 28.4 months, Western region 25.4 months, and Southwestern at 15.2 months (*p* < 0.001) (Fig. 2).

**Fig. 3:**
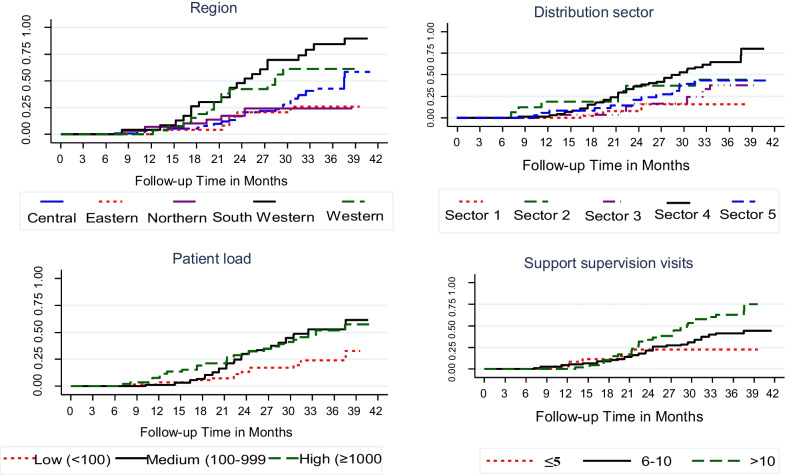
Kaplan–Meier curve comparing time to gold ranking of health facilities in key factors, Uganda 2011–2016. Kaplan–Meier curve comparing time to gold ranking of health facilities stratified by region, distribution sector, patient load and support visits in the procurement and supply chain strengthening project (PSSP), Uganda 2011–2016

Cumulative probability of achieving a gold status was 52.7% at 41.5 months. This was significantly different when stratified by distribution sector (*p* < 0.001), region (*p* < 0.001), patient load (*p* = 0.008) and number of RFSO visits (*p* = 0.004) (Fig. 3).

Multivariate analysis showed that distribution sector, HF level, patient load, number of support supervision visits, HF start year and preliminary assessment score were all significant predictors of gold status (Table 3).

**Table 3:** Factors associated with achieving a gold ranking in the health facilities, Uganda 2011–2016

Variables	All	Gold	cHR	*p*-value	aHR (*n* = 190)	*p*-value
Total	216	83				
Region (*n* = 216)	*n* (%)	*n* (%)				
Central	102 (47.2)	35 (42.2)	Reference			
Eastern	29 (13.4)	6 (7.2)	0.55	0.178		
Northern	31 (14.4)	7 (8.4)	0.52	0.12		
South Western	24 (11.1)	20 (24.1)	2.98	< 0.001		
Western	30 (13.9)	15 (18.1)	1.9	0.038		
Distribution sector (*n* = 216)						
Sector 1	44 (20.4)	6 (7.2)	Reference		Reference	
Sector 2	17 (7.9)	7 (8.4)	3.38	0.029	4.36	0.012
Sector 3	35 (16.2)	10 (12.1)	2.13	0.144	1.72	0.315
Sector 4	83 (38.4)	47 (56.6)	5.72	< 0.001	4.11	0.002
Sector 5	37 (17.1)	13 (15.7)	3	0.026	3.69	0.014
HF level (*n* = 216)						
HC II	1 (0.5)	0 (0)	Reference		Reference	
HC III	136 (63)	43 (51.8)	0.83	0.533	2.03	0.152
HC IV	11 (5.1)	7 (8.4)	1.67	0.266	4.70	0.01
Hospital	37 (17.1)	19 (22.9)	1.26	0.507	1.60	0.217
Special clinic	31 (14.4)	14 (16.9)	1.00	–	1.00	–
Patient load (*n* = 194)						
Low volume (< 100)	55 (25.5)	13 (15.7)	Reference		Reference	
Medium volume (100–999)	87 (40.3)	43 (51.8)	2.46	0.005	2.19	0.026
High volume (≥ 1000)	52 (24.1)	27 (32.5)	2.46	0.008	2.97	0.034
Missing	22 (10.2)	0 (0)	–			
Support supervision visits (*n* = 198)						
≤ 5	38 (17.6)	7 (8.4)	Reference		Reference	
6–10	113 (52.3)	44 (53)	1.5	0.319	3.33	0.024
> 10	47 (21.8)	32 (38.6)	2.83	0.013	5.78	0.003
Missing (no visits)	18 (8.3)	0 (0)	–			
Status (*n* = 216)						
Active	205 (94.9)	80 (96.4)	Reference			
Closed	1 (0.5)	0 (0)	1.00	–		
Not active	10 (4.6)	3 (3.6)	1.77	0.335		
HF category (*n* = 216)						
Accredited	194 (89.8)	83 (100)	Reference			
Accredited (closed)	1 (0.5)	0 (0)	1.00	–		
For accreditation	21 (9.7)	0 (0)	0	1		
Start year (*n* = 198)						
2013	152 (70.4)	68 (81.9)	Reference		Reference	
2014	36 (16.7)	13 (15.7)	1.49	0.204	4.19	< 0.001
2015	7 (3.2)	2 (2.4)	5.04	0.033	31.58	< 0.001
2016	3 (1.4)	0 (0)	1.00	–	1.00	–
Missing	18 (8.3)	0 (0)	–			
Preliminary assessment score (*n* = 194)						
< 50	13 (6)	3 (3.6)	Reference		Reference	
50–69	44 (20.4)	18 (21.7)	1.62	0.44	2.36	0.186
70–89	106 (49.1)	41 (49.4)	1.55	0.465	1.91	0.303
≥ 90	31 (14.3)	20 (24.1)	3.45	0.046	4.58	0.021
Missing	22 (10.2)	1 (1.2)	–			

Factors associated with achieving a gold ranking in the health facilities evaluated on health system strengthening in the procurement and supply chain strengthening project (PSSP), Uganda 2011–2016

**Fig. 4:**
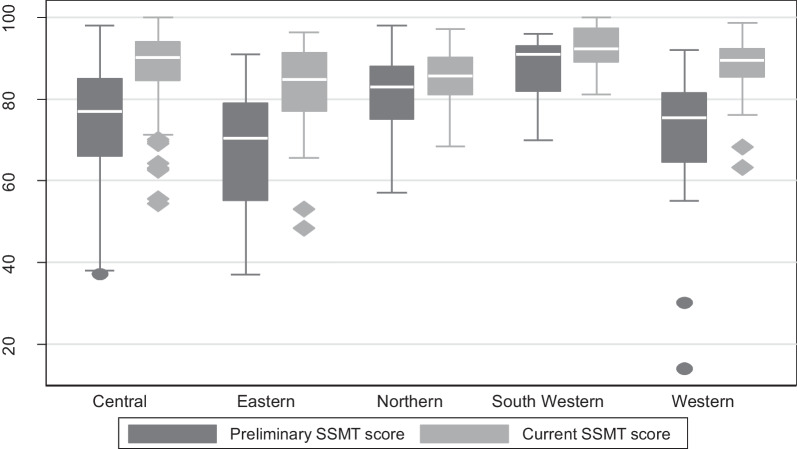
Average baseline Support Supervision Monitoring Tool (SSMT) scores and intervention follow-up SSMT scores stratified by region

There was a significant improvement from 79 to 91% (*p* < 0.001) in inventory accuracy for on-hand inventory at the end of the reporting period. A total of 929 health workers were trained in LMIS and SCM, and 1575 support supervision visits were also conducted (Table 4). The average SSMT scores increased between baseline and at the end of the reporting period for all regions (Fig. 4).

**Table 4:** Assessment indicators showing baseline and end line scores

Indicator	Baseline score	End line score	*p*-value
Percentage of facilities with adequate stock (A)	54%	71%	0.002
Inventory accuracy (B)	79%	91%	< 0.001
Average stock in levels (C)	54%	96%	< 0.001
Order fill rate (D)	93%	97%	0.375
Facility reporting rates (E)	91%	100%	< 0.001
Number of support visits and personnel trained (F)	929 health workers trained and 1575 support supervision visits

### FROST and GIS

Five hundred and twenty-two stock redistributions had been authorized in FROST as of August 2016 with 162 (31%) in 2014, 235 (45%) in 2015 and 125 (24%) in 2016. A total of 308 (59%) were stock redistributions of ART medicines, 82 (15.7%) of laboratory equipment, 75 (14.4%) of OIs medication and 57 (10.9%) of HIV test kits. One of the major goals of FROST was to facilitate redistributions between regions; and since its inception, 8% of redistributions have fallen in this category.

We also saw an improvement in the percentage of facilities with adequate stock levels of indicator commodities from 54% (191 HF) to 71% (215 HF) which was statistically significant (*p* = 0.002) as well as average percentage stock in levels at the facilities (54% to 96%, *p* < 0.001) over the same period (Table 4).

### WhatsApp^®^

With the introduction of WhatsApp^®^ messenger, we saw a statistically significant improvement in facility reporting (91% (191 HF) to 100% (215 HF), *p* < 0.001) as well as a slight improvement in order fill rate in terms of customer (93% to 97%, *p* = 0.375) which was not statistically significant (Table 4).

## Discussion

We implemented four interventions MaTHSS, FROST, GIS and the use of WhatsApp^®^ messenger at the health facility level in an attempt to improve SCM indicators. Whereas SCM is affected by a number of factors, including transportation infrastructure, our findings suggest that with innovative facility level interventions, improvements are possible. Improvements in national infrastructure typically take longer to implement and therefore innovative solutions at the last mile (at facility level) are needed. Our findings suggest that support supervision plays a major role in improving stock management at facility level. Our use of RFSOs (who are all pharmacists) to train HF staff allowed for task shifting as we were able to use the trained HF staff on the ground and utilize the RFSOs for technical support at multiple sites. Our end goal is to sustain high performance (quality service provision) with supervision visits slowly removed due to cost implications. It will be important to assess sustainability given that a similar study found that sites revert back to baseline performance once interventions are discontinued [16].

The difference in time to silver status ranking by region suggests that clinics in different regions were varyingly receptive to support supervision. However, without facility level data it was hard to tease out what the main drivers for these differences were. Given the different numbers of health facilities in each region, it is feasible that this could also account for some of the difference. Furthermore, given that health facilities characteristics varied by region on variables like the average preliminary SSMT score; this could further explain these variances. It is not surprising that sites with multiple visits were more likely to reach gold status given the dependence on multiple visits to achieve gold ranking.

As a result of these interventions, we saw a rise in percentage of HF with accurate stock card inventory from 79 to 91%; adequate stock levels from 54 to 71% and stock reporting rates from 91 to 100%. We also saw an improvement in the order fill rate from 93 to 97%. Most importantly, we saw a significant reduction in HF reporting stock-outs from 46 to 4% in the analysis period. Our findings also suggest that field-based stock management (FROST) can greatly reduce stock-outs at facility level. In addition, FROST allowed for quicker decision-making as data were real time and stock redistributions could be authorized on the same day as the need was identified. The tool also played a major role in reducing loss due to expiries as short-dated medicines were given highest priority during redistributions. Furthermore, the possibility of stock redistribution between regions was impactful. Before FROST, each region had its own tool, every site needed to be visited before stock redistribution decisions could be made, the old tools updated manually, and past information on distribution was unavailable which made the process quite long and unnecessarily complicated.

Stock-outs remain a pertinent issue since we were unable to eliminate them. We must continue to minimize stock-outs because we know that both the HIV treatment cascade and patient outcomes are adversely impacted [17, 18]. We found that other factors played a major role in a facility reporting a stock-out. Firstly, high staff turnover particularly of trained staff especially at lower-level HF affected the quantification process. We also found a problem with fluctuating patient numbers due to high patient mobility, in which case, HF with high numbers of patient transfer-in were also more likely to report a stock-out.

Furthermore, the use of WhatsApp^®^ and mobile technology greatly improved our outcomes by improving the timeliness of order delivery. Another study has had similar findings using SMS [16].

### Strengths and limitations

A major strength of this analysis was the high completeness level of data which were routinely collected from all 216 sites. The interventions were implemented in both rural and urban HF covering a wide geographical area. The findings give a fair representation of the national picture and therefore may be relevant to SCM programs in countries with similar settings.

Given the high financial investment required, routine implementation of these interventions may not be feasible for programs in resource limited settings like sub-Saharan Africa. One valuable, low-cost method that can be implemented cheaply was the readily available smart phone app to improve reporting and transfer of relevant information for decision-making. Results from this evaluation provide proof that if correct interventions are applied, improvement in SCM program performance can be achieved. We also acknowledge the possibility that these analyses were affected by temporal trends.

## Conclusions

Health system strengthening and mentorship is feasible at health facility level. It is however only possible if the relevant human resources and financial investment in infrastructure are available to ensure its smooth implementation. SCM programs should endeavor to link data from different facilities to improve stock redistribution potential. Also, the use of GIS can further simplify the stock enumeration process. Furthermore, in cases where internet access is not ubiquitous or reliable, SCM programs should consider the potential of readily available smart phone apps to improve reporting and transfer of relevant information.

### Recommendations

To achieve the UNAIDS 90-90-90 goals in sub-Saharan Africa, limited resources will have to be distributed among a myriad of competing needs. We must use the available resources efficiently; encourage best practices and minimize waste. Last mile interventions can vastly improve SCM with much less investment needed.

## Data Availability

The datasets generated and analyzed during the current study are not publicly available because of proprietary reasons but a minimum dataset that can be used to replicate the study findings is available from the corresponding and lead author (sowedi.muyingo@medicalaccess.co.ug) or from one of the co-authors (davidetoori@gmail.com) on reasonable request.
